# Acute Appendicitis in Situs Inversus Totalis: A Case Report

**DOI:** 10.7759/cureus.22947

**Published:** 2022-03-08

**Authors:** Dharmendra K Pipal, Vibha Rani Pipal, Seema Yadav

**Affiliations:** 1 General Surgery, All India Institute of Medical Sciences, Gorakhpur, Gorakhpur, IND; 2 Obstetrics and Gynecology, All India Institute of Medical Sciences, Gorakhpur, Gorakhpur, IND; 3 Anaesthesia, Rajmata Vijaya Raje Scindia Medical College, Bhilwara, IND

**Keywords:** abdominal ct, midgut malrotation, laparoscopic appendicectomy, situs inversus totalis, left-sided acute appendicitis

## Abstract

Left-sided acute appendicitis (LSAA) is a rare cause of acute pain in the abdomen and is associated with developmental anomalies such as situs inversus (viscus) totalis (SIT) and midgut malrotation (MM). Due to the rarity along with the atypical presentation, diagnosis of LSAA is difficult, and if it is not managed timely, complications of appendicitis such as perforation can result. Imaging including contrast-enhanced CT scans and ultrasound aids in establishing the diagnosis. In case of a diagnostic dilemma, a diagnostic laparoscopy is an optimal option that offers diagnostic as well as therapeutic benefits. Operative intervention, preferably laparoscopic, is the standard treatment of LSAA. We report a case of appendicitis in a 36-year-old man with SIT detected radiologically who presented with pain in the left side of the lower abdomen for two days. Minimal tenderness was noted on the left iliac fossa during per abdominal examination. Abdominal ultrasonography was showing probe tenderness in the left iliac fossa, and contrast CT of the abdomen was suggestive of appendicitis with SIT. The patient was managed by laparoscopic appendicectomy. Therefore, we conclude that LSAA should be listed in the differentials of the various causes of left-sided pain in patients with SIT or MM. Clinical diagnosis is often difficult, and CT scan is crucial to establish the diagnosis as well as confirm rotational anomalies. Surgery, preferably laparoscopic, represents the appropriate treatment of LSAA.

## Introduction

Acute appendicitis is one of the common surgical conditions where patients require immediate surgical intervention to avoid complications of its perforation. Additionally, almost 4%-8% of all patients visiting emergency actually suffer from acute appendicitis [1]. Situs inversus totalis (SIT) is a rare reverse positional disorder of both thoracic and abdominal organs, with an incidence of 1 in 10,000-50,000 persons [2-6].

As appendicitis causes right-sided pain, the clinician does not consider it as a differential diagnosis of acute appendicitis if the patient presents with left-sided lower abdominal pain. Left lower quadrant pain can be due to various gastrointestinal causes such as acute colonic diverticulitis, intestinal obstruction or perforation, complicated hernia, gastroenteritis, and appendicitis including both right- and left-sided. Genitourinary causes can manifest as left-sided, pain such as renal/ureteric colic, epididymo-orchitis, prostatitis, and testicular torsion.

This may confuse the treating clinician, which may cause appendicular perforation [7]. Therefore, sonography and CT of the abdomen are very crucial noninvasive modalities to diagnose the condition and to prevent complications.

## Case presentation

**Figure 1:**
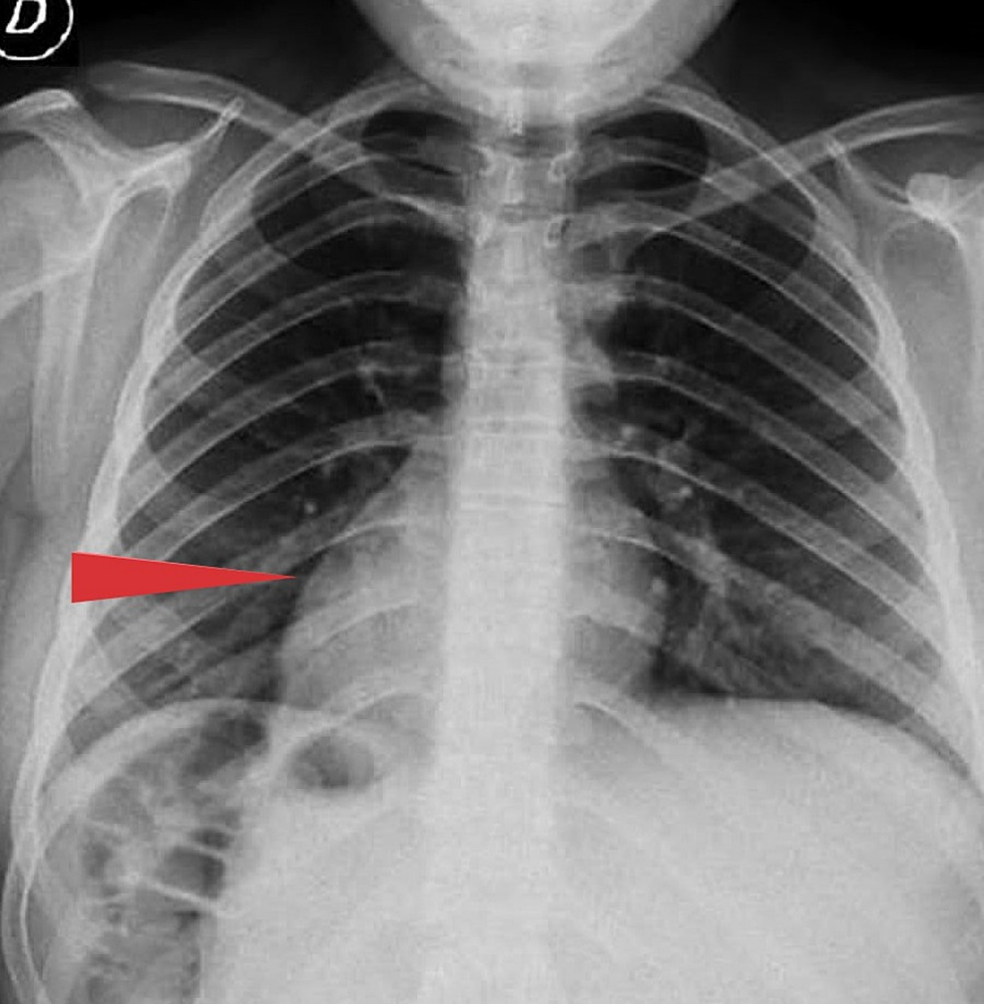
Chest radiograph showing dextrocardia

**Figure 2:**
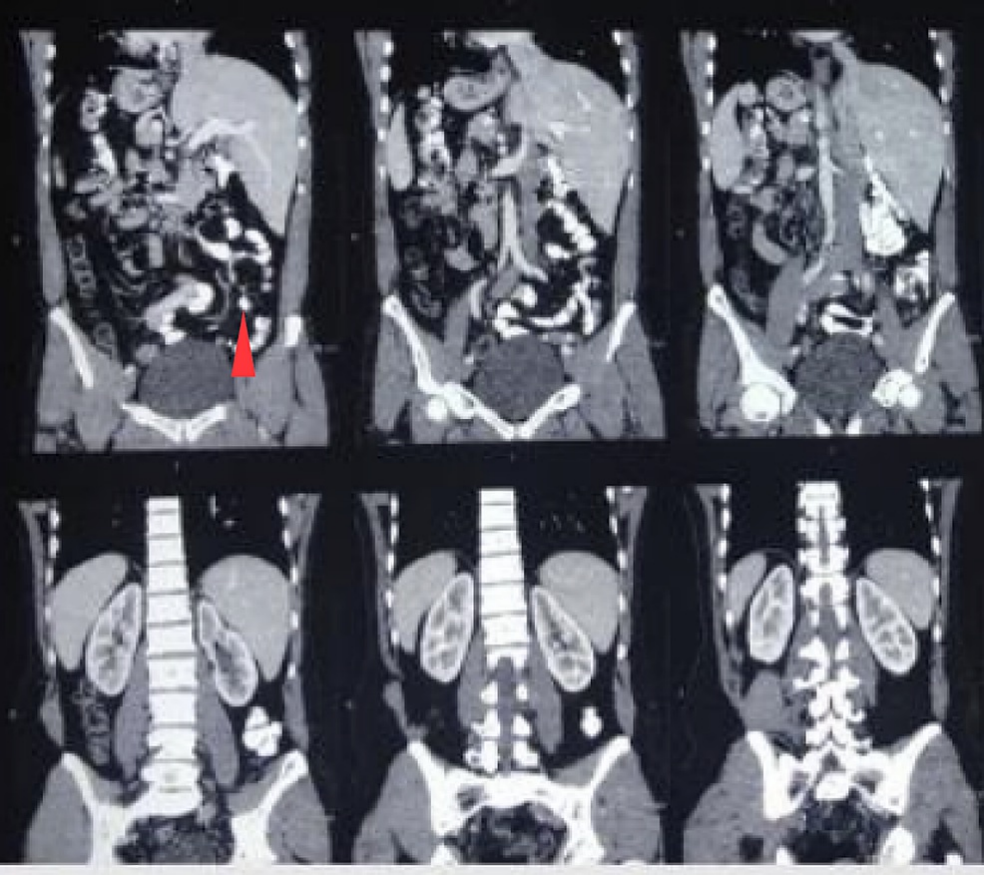
CT of the abdomen confirming SIT and appendicitis (red arrow) SIT, situs inversus totalis

A 36-year-old man with SIT detected radiologically presented with pain in the left side of the lower abdomen for two days. He was not having any urinary complaints. Minimal tenderness was noticed on the left iliac fossa during per abdominal examination. Blood investigations revealed a higher value of total leucocyte count (11000 × 10^9^/L) with neutrophilic predominance accounting for 80%. Liver function and renal function tests were within the normal range. Cardiac shadow was directed on the right side of the chest radiograph (Figure 1). Abdominal ultrasonography showed probe tenderness in the left iliac fossa, and contrast CT of the abdomen (Figure 2) was suggestive of appendicitis with SIT. Therefore, diagnostic laparoscopy with appendicectomy was planned.

As the patient was a known case of SIT, the monitor was placed on the left side of him. The first 10-mm port camera port was introduced supra-umbilically with Hassan's technique. The second 5-mm port was placed under vision supra-pubically and the other 5-mm port was placed in the right iliac fossa as working ports.

**Figure 3:**
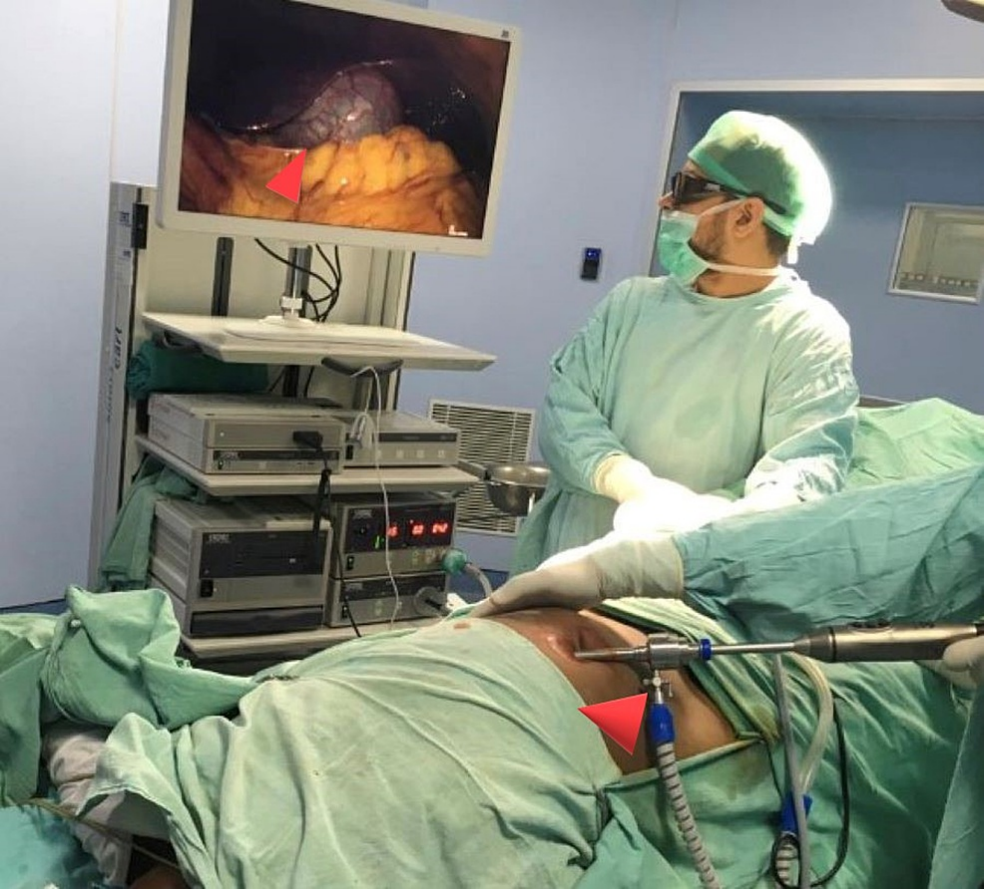
Liver and gall bladder visualized on the left side as evident by directing telescope on the left side

**Figure 4:**
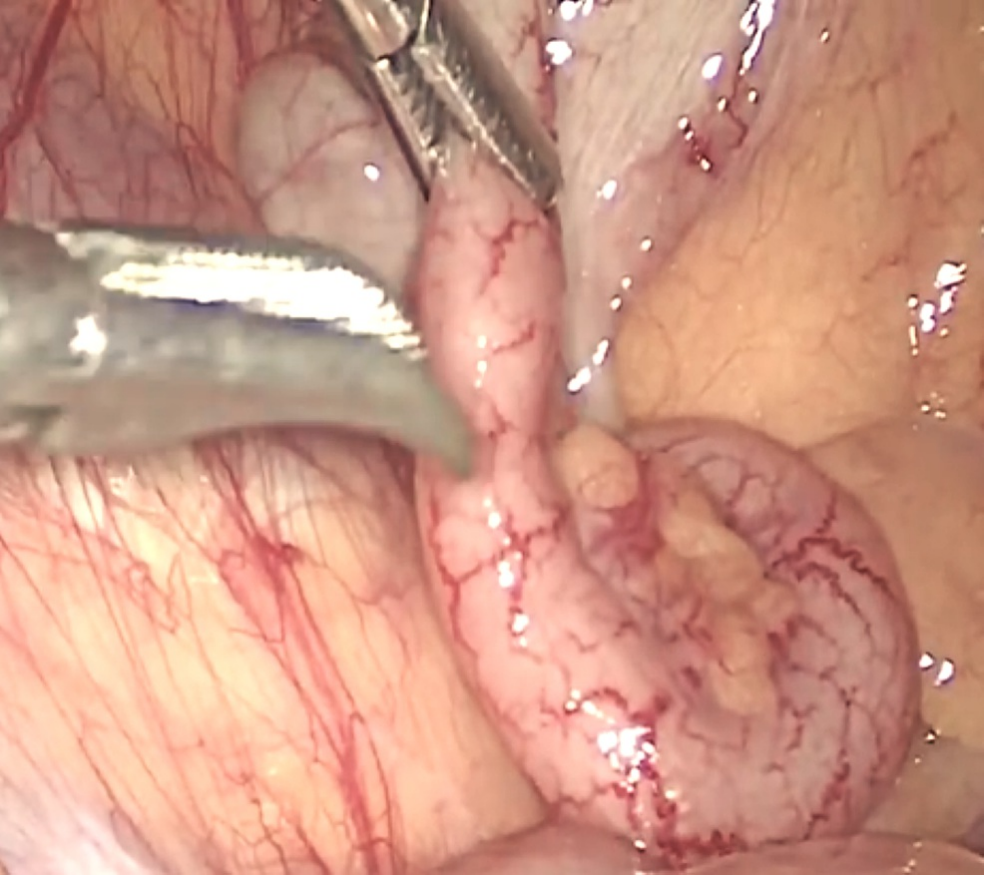
Intraoperative image showing inflamed appendix

Intraoperative findings had coincided with ultrasonography and CT scan findings, liver and gall bladder were positioned on the left side (Figure 3), and the greater curvature of the stomach was on the right side. The whole abdomen was thoroughly examined. Solid organs were normal-appearing, no free fluid was there, bilateral subdiaphragmatic spaces and Morrison’s pouch were clear, omentum and gut loops were also normal, and no Meckel's like diverticulum was found. The appendix exactly mirrored its normal right-sided position, i.e., present in the left iliac fossa and was appearing minimally inflamed and thus it was removed (Figure 4). The histopathological report confirmed acute appendicitis. During the postoperative period, the patient’s recovery was uneventful.

## Discussion

Situs inversus (SI) is an autosomal recessive disease characterized by the congenital inversion of abdominal viscera and called SIT on the transposition of both abdominal and thoracic viscera [3,4]. The pain of appendicitis begins around the periumbilical region and migrates to the right lower quadrant. It is an acute abdominal condition that warrants immediate operative interventions to prevent complications such as perforation [2,7].

There are so many causes of left iliac fossa pain that include colonic diverticulitis, Meckel’s diverticulitis, strangulated hernia, bowel obstruction, psoas abscess, and right- and left-sided acute appendicitis (LSAA), in female patients, causes including left ovarian disease, pelvic inflammatory disease or twisted/ruptured ovarian cyst may present as left-sided abdominal pain [2,6,8]. In a normal individual, the right-sided lower quadrant appendix is a result of 270° counterclockwise rotation of the midgut loop and when the rotation takes place 270° clockwise, the end result is the SI. Similarly, when the midgut loop, either does not rotate or incompletely rotates around the long axis of the superior mesenteric artery, the end result is midgut malrotation [2]. The reverse position of the abdominal viscera in SIT leads to left lower-quadrant pain in acute appendicitis, which creates a diagnostic dilemma. Akbulut et al. reported that while 62% of cases present with pain on the left and 14% on the right side of the lower abdomen, approximately 7% of patients have bilateral lower abdominal pain [2,5,7,8]. As the nervous system sometimes may not follow the visceral malrotation, atypical pain location creates diagnostic confusion to the clinicians, and approximately half of the patients can be precisely diagnosed clinically as acute appendicitis preoperatively [5,7].

A thorough physical examination, followed by radiographic imaging of the chest and abdomen and electrocardiography, can identify most cases of SI. A chest radiograph that indicates dextrocardia, and an abdominal CT scan describe visceral transposition [6,7]. Due to easy availability, ultrasonography is a commonly used imaging modality, but it is highly radiologist-dependent. CT is highly sensitive to diagnose appendicitis in rotational anomalies such as SIT or MM [7,9-12].

Contini et al. first reported the role of laparoscopy in a patient SIT with appendicitis [7,12] in 1997, but the technical procedure was not described. However, laparoscopy is technically more challenging because of the reverse view of abdominal organs in a patient of SIT, and in spite of that, it is indicated for diagnosis as well as for the management of acute appendicitis in a patient of SIT [9]. Regarding the port sites, Golash [7,10,12] used a 10-mm port in the left iliac fossa and a 5-mm port in the suprapubic region as working ports. Palanivelu et al. introduced a 10-mm supra-pubic port as right-hand manipulation and a 5-mm umbilical telescopic port in a patient with left-sided appendicitis. No standard port positions are yet defined, and they adopted a tailored approach for port placements [7,11,12].

We used the umbilical port for telescopic introduction along with the right iliac port as a left-hand port and the suprapubic one as a right-hand port. This is a mirror image of the “two-handed” technique that is used for routine laparoscopic appendectomy. Therefore, laparoscopy plays a significant role in diagnosis and management of a case of acute appendicitis associated with SIT [10-12].

## Conclusions

A patient with left-sided lower abdominal pain along with dextrocardia on chest radiograph should have a suspicion of acute appendicitis, and further workup in the form of contrast CT of the abdomen must be carried out to establish the diagnosis of appendicitis as well as situs anomaly. Laparoscopy is challenging due to the reverse anatomy of the abdominal viscera but is still considered as a procedure of choice in a patient of SIT.
